# A Walking Intervention Supplemented With Mobile Health Technology in Low-Active Urban African American Women With Asthma: Proof-of-Concept Study

**DOI:** 10.2196/13900

**Published:** 2020-03-11

**Authors:** Sharmilee M Nyenhuis, Guilherme Moraes Balbim, Jun Ma, David X Marquez, JoEllen Wilbur, Lisa K Sharp, Spyros Kitsiou

**Affiliations:** 1 Department of Medicine University of Illinois at Chicago Chicago, IL United States; 2 Department of Kinesiology and Nutrition University of Illinois at Chicago Chicago, IL United States; 3 Department of Women, Children and Family Nursing Rush University Chicago, IL United States; 4 Department of Pharmacy Systems, Outcomes and Policy University of Illinois at Chicago Chicago, IL United States; 5 Department of Biomedical and Health Information Sciences University of Illinois at Chicago Chicago, IL United States

**Keywords:** activity trackers, text message, physical activity, asthma, African-American, women, mHealth, smartphone, mobile phone

## Abstract

**Background:**

Physical inactivity is associated with worse asthma outcomes. African American women experience disparities in both physical inactivity and asthma relative to their white counterparts. We conducted a modified evidence-based walking intervention supplemented with mobile health (mHealth) technologies to increase physical activity (PA).

**Objective:**

This study aimed to assess the preliminary feasibility of a 7-week walking intervention modified for African American women with asthma.

**Methods:**

African American women with suboptimally controlled asthma were identified from a health system serving low-income minorities. At a baseline data collection visit, participants performed spirometry and incremental shuttle walk test, completed questionnaires, and were given an accelerometer to wear for 1 week. The intervention comprised an informational study manual and 3 in-person group sessions over 7 weeks, led by a nurse interventionist, in a community setting. The supplemental mHealth tools included a wearable activity tracker device (Fitbit Charge HR) and one-way text messages related to PA and asthma 3 times per week. A secure Web-based research platform, iCardia, was used to obtain Fitbit data in real time (wear time, moderate-to-vigorous physical activity [MVPA] and sedentary time) and send text messages. The feasibility of the intervention was assessed in the domains of recruitment capability, acceptability (adherence, retention, engagement, text messaging, acceptability, complaints, and concerns), and preliminary outcome effects on PA behavior (change in steps, duration, and intensity).

**Results:**

We approached 22 women, of whom 10 were eligible; 7 consented, enrolled and completed the study. Group session attendance was 71% (5/7), 86% (6/7), and 86% (6/7), respectively, across the 3 sessions. All participants completed evaluations at each group session. The women reported being satisfied or very satisfied with the program (eg, location, time, and materials). None of them had concerns about using, charging, or syncing the Fitbit device and app. Participants wore their Fitbit device for at least 10 hours per day in 44 out of the 49 intervention days. There was an increase in Fitbit-measured MVPA from week 1 (19 min/week, SD 14 min/week) to the last week of intervention (22 min/week, SD 12 min/week; Cohen *d*=0.24, 95% CI 0.1 to 6.4). A slight decrease in step count was observed from week 1 (8926 steps/day, SD 2156 steps/day) to the last week of intervention (8517 steps/day, SD 1612 steps/day; Cohen *d*=−0.21, 95% CI −876.9 to 58.9).

**Conclusions:**

The initial feasibility results of a 7-week community-based walking intervention tailored for African American women with asthma and supplemented with mHealth tools are promising. Modifications to recruitment, retention, and the intervention itself are needed. These findings support the need to conduct a further modified pilot trial to collect additional data on feasibility and estimate the efficacy of the intervention on asthma and PA outcomes.

## Introduction

### Background

Asthma is a highly prevalent chronic disease that affects 1 in 12 people in the United States [1]. However, asthma disproportionately impacts African American women [1]. African American women have higher rates of asthma exacerbations and health care utilization, worse lung function, poorer asthma-related quality of life, and a higher crude asthma mortality rate compared with white women [1-3].

Research to date shows that engaging in regular, moderate physical activity (PA) improves asthma-related quality of life and asthma control and decreases asthma health care utilization [4-6]. Despite the benefits of PA in asthma and guideline recommendations to engage in PA, individuals with asthma, particularly women, are less likely to engage in PA than women without asthma (odds ratio [OR] 3.66 vs 4.37) and men with asthma (OR 3.66 vs 4.57) [7-10]. Given the connection between poor asthma outcomes and physical inactivity, addressing PA among sedentary African American women with asthma is imperative [11,12].

Of the existing PA interventions tested in people with asthma, only 2 have focused on walking. The first was a long-term (1 year) community-based study but was not a randomized controlled trial; the second was a 12-week randomized controlled trial in an academic center’s fitness center [5,13]. Both studies demonstrated the safety of walking interventions in asthma and improvements in asthma control or quality of life but included predominately white women (>50%). As promising as these limited results are, no PA interventions to date have tailored to the needs of African American women with asthma.

Although African American women with asthma are faced with many of the same barriers to PA as other women with and without asthma, this population faces unique barriers. Specifically, the barriers identified among African American women, in general, include a lower self-efficacy for PA, lack of social support, culturally based preferences for body type and hair, community safety concerns, lack of sidewalks, and a lack of physically active role models [7,14-17]. All of these barriers combined make taking on a new behavior such as PA in the face of a chronic disease difficult. Research supports the development of tailored PA interventions in African American women as there is a need to consider the particular needs of African American women, which is a missing component in current PA interventions for asthma [12,18].

The Women’s Lifestyle Physical Activity Program, a group-based behavioral intervention developed for low-active urban African American women, found an improvement in daily steps and high adherence to the walking program [19]. Although this program was tailored for African American women, an exclusionary criterion was chronic pulmonary disease. Using feedback we obtained from African American women with asthma who examined materials from the Women’s Lifestyle Physical Activity Program [16], we tailored a PA intervention to meet their needs specific to asthma and lifestyle preferences. The modifications included an asthma education session, led by an asthma educator, the use of mobile health (mHealth) tools such as SMS (to deliver advice, reminders, and motivational support to increase walking), and the utilization of commercial wearable activity tracker devices (Multimedia Appendix 1) [19,20]. Technology-based interventions have a high level of user satisfaction, especially among ethnic and racial minorities and urban or low-income individuals with asthma and have been used to promote PA in African American women without asthma [21-25].

### Objective

The objective of this study was to assess the feasibility (recruitment, retention, adherence to group meetings, and adherence to Fitbit device wear time) of the modified 7-week walking intervention (ACTION) and assess preliminary outcome effects on PA behavior (change in steps, duration, and intensity).

## Methods

### Design

This proof-of-concept study was a prepilot test of the ACTION study funded by the National Heart, Lung, and Blood Institute. The aim of this prepilot study was to recruit up to 10 African American women with asthma over a period of 3 months to test and refine the study procedures and materials, including recruitment methods and content of the ACTION intervention.

### Setting and Participants

Participants were recruited from a large urban medical center serving approximately 60% African Americans. Potential participants were approached in a subspecialty asthma clinic after being informed by their physician of the study and agreeing to be contacted or were previously screened for asthma studies and provided consent to be contacted for future studies. Participants were screened in-person or by phone and were eligible for the study if they had a physician diagnosis of asthma, self-identified as African American and female, were between the ages of 18 and 70, registered as a patient at the medical center, were low-active as defined by self-report of <150 min of moderate to vigorous PA per week (“On average, how many minutes per week of moderate to vigorous intensity physical activity do you engage in?”), and had suboptimally controlled asthma (Asthma Control Test score <20) [26]. If eligible, the woman was invited to participate in the study. Interested participants came for a baseline assessment visit at the Clinical Research Center at the University of Illinois at Chicago, where written informed consent was obtained from each participant before any study procedures. All participants received US $25 in remuneration for their time and travel after completion of the baseline assessment visit and at the last intervention group session (total US $50). The study (protocol #2016-0466) was approved by the University of Illinois at Chicago Institutional Review Board.

### Procedures

Participants attended a baseline assessment visit approximately 1 to 3 weeks before the start of the intervention (Multimedia Appendix 2). The visit consisted of informed consent procedures, baseline data collection pertaining to demographics, PA and asthma-related study measures (spirometry without bronchodilation, incremental shuttle walk test [ISWT], Asthma Control Questionnaire [ACQ], and Asthma Quality of Life Questionnaire [AQLQ]), provision of a research-grade activity monitor (ActiGraph GT3X+), download of the Fitbit mobile app on participant’s mobile device (eg, smartphone or tablet), and pairing process of the Fitbit activity tracker device with the mobile app. After pairing the Fitbit device with the participant’s mobile device, the Fitbit device was kept in the possession of the study team until the first group visit and given to the participant at that time to begin wearing. Participants were asked to wear the research-grade activity monitor on their nondominant wrist for 7 consecutive days during their waking and sleeping hours, except when bathing. A wrist-based accelerometer was used to enhance wear compliance, which has been observed by the 2011-2013 National Health and Nutrition Examination Survey group [27]. The 7-week intervention began after all participants completed the baseline data collection, including wearing the accelerometer. We collected the feasibility and acceptability of the intervention both during and/or after completion of the intervention, when applicable (Table 1).

**Table 1:** Feasibility metrics, assessment strategy and methods used, and timepoint measured.

Feasibility metric and assessment strategy		Assessment method	Timepoint measured
**1. Process recruitment capability**			
	1.1. Recruitment length, rate, and eligibility criteria	Recruitment length was reported as the length of time it took to recruit the desired sample size. Recruitment rates were reported as the number of people approached (phone or in-person), screened, eligible, and not eligible. Reasons for ineligibility were recorded if applicable.	Weeks –2 to 0
	1.2. Recruitment strategies and barriers	Recruitment barriers were identified by research staff and recorded in field notes and transferred to a Microsoft Excel sheet. Recruitment barriers and strategies were reviewed by the research team at weekly meetings.	Weeks –2 to 0
**2. Feasibility: acceptability and suitability**			
	2.1. Adherence	Adherence was calculated as a percentage of sessions attended. Adherence to wearing the Fitbit device was measured as wear time using Fitbit data. Adherence to step goals was measured as percentage of days in a week that the step goal was met by participants using Fitbit data.	Weeks 1 to 7
	2.2 Retention	Retention rate was calculated as the number of participants who completed the postintervention evaluation subtracted from those who were enrolled in the intervention. Participants were categorized into 2 groups: those who completed ≥2 group sessions and used technology components, and those who wore the Fitbit device during the intervention and received SMS^a^ but attended <2 group sessions.	Weeks 1 to 7
	2.3. Engagement	Participant engagement was assessed by the same nurse interventionist at each group session using a 7-point Likert scale with 1=not at all engaged and 7=very engaged.	Weeks 1, 3, and 7
	2.4. Text messages delivery and content appropriateness	Text message delivery was measured as the number of SMS successfully delivered in relation to the number of planned SMS to be sent.	Weeks 1 to 7
		Content appropriateness was assessed by feedback provided by participants using a 5-point Likert scale with 1=not at all appropriate to 5=very appropriate.	Weeks 1, 3, and 7
	2.5. Acceptability	Location, time, text message, Fitbit (device, app, and syncing), and overall program acceptability. Overall satisfaction was measured using either a 5-(text message) or 7-point (location, time, Fitbit, and overall program) Likert scale with 1=not at all satisfied to 7=very satisfied. We used 7-point Likert scales for satisfaction (1 representing not satisfied and 7 representing very satisfied) of (1) intervention location, (2) time of intervention sessions, (3) Fitbit (charging, syncing, and app use), and (4) overall intervention.	Week 7
	2.6. Complaints and concerns	Complaints and concerns about the program were recorded when participants reported to the research team or by written feedback.	Weeks 1 to 7
**3. Preliminary outcome effects (PA^b^ and asthma): assessments of the safety, compliance, and treatment effect of the study.**			
	3.1. Adverse events and serious adverse events	Participants were asked about minor events at each session and they were recorded in the REDCap database.	Weeks 1 to 7
	3.2. Participants demographic/clinical data	Demographic data were obtained via a self-report questionnaire at the baseline. Baseline clinical data (asthma and physical activity measures) included ACQ^c^ [38], mini-AQLQ^d^ [39], FEV_1_^e^ % predicted, mean daily steps per day measured by ActiGraph GT3X+, and ISWT^f^.	Week 0
	3.3. Preliminary outcomes effects	Primary outcome of exercise was daily step count from Fitbit raw data. Secondary outcomes from Fitbit included time spent in moderate-to-vigorous physical activity, termed by Fitbit as *active minutes* at ≥3 METs^g^, and sedentary time (≤1 METs)	Weeks 1 to 7

^a^SMS: short message service.

^b^PA: physical activity.

^c^ACQ: Asthma Control Questionnaire.

^d^AQLQ: Asthma Quality of Life Questionnaire.

^e^FEV_1_: forced expiratory volume in 1 second.

^f^ISWT: incremental shuttle walk test.

^g^MET: metabolic equivalent.

### Intervention

The Women’s Lifestyle Physical Activity Program is a 48-week evidence-based moderate-intensity walking intervention designed for urban low-active African American women [19]. The intervention used social cognitive theory strategies that were applied systematically throughout to target specific barriers that African American women face when engaging in PA, including lack of social support, self-efficacy, and environmental barriers. The intervention components included a tailored walking prescription, a PA self-monitoring tool (pedometer), group discussions to address goals and barriers, and motivational telephone calls (automated vs personal). We adapted this intervention using the Behavior Change Wheel and Theoretical Domains Framework to address the unique barriers to PA experienced by urban low-active African American women with asthma [16,20,28]. The adaptations included a 45-min dedicated asthma education session (based on the American Lung Associations Asthma Basics course) at the start of the intervention, motivational text messages, group sessions with African American women with asthma, an optional walking session, and informational written materials on exercising with asthma. Women participated in the modified 7-week intervention (ACTION) after completing baseline data collection. The intervention consisted of (1) self-monitoring and self-regulation of PA through the use of a wrist-based wearable activity tracking device (Fitbit Charge HR) and Fitbit mobile app, (2) PA goal-setting, (3) in-person group sessions, and (4) text messages (Multimedia Appendix 1).

**Figure 1:**
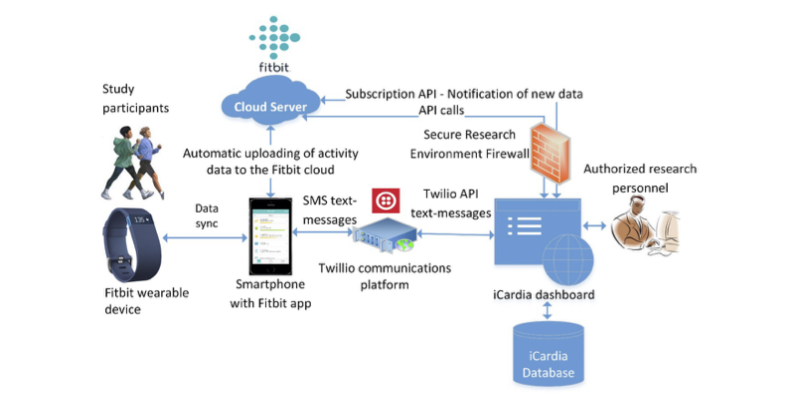
iCardia platform.

Intervention data were managed through iCardia, a secure research platform that enables remote collection of patient-generated health data from multiple connected health devices, including Fitbits, through the use of Web-based application programming interfaces (APIs) [29]. iCardia comprises user-friendly visualization and data exporting tools that allow authorized study researchers to view and analyze objectively measured Fitbit data pertaining to PA (eg, step count, PA intensity, type, and duration) and sedentary behavior. iCardia’s dashboard presents all incoming data to researchers in the form of graphs and allows them to send text messages to participants’ mobile phones. Study participants did not have access to iCardia; they only interacted with their Fitbit mobile app and PA monitor. The text messaging feature is integrated with the iCardia clinical dashboard and utilizes Twilio’s API to send messages via the SMS protocol. In the context of this study, iCardia was used to facilitate remote monitoring of PA measures from Fitbit devices and delivery of text messages each week to increase PA levels in patients with asthma (Figure 1). Text message content is described in more detail below.

### Self-Monitoring of Physical Activity With Fitbit Charge HR

Fitbit Charge HR is a reliable and user-friendly tracker that collects a wide range of PA and biological data, including step count, PA intensity (*light*, *fairly active*, and *very active*), sedentary time (any waking behavior characterized by an energy expenditure ≤1 metabolic equivalents [METs] while in sitting or lying posture), calories burned, sleep, and continuous heart rate [30-33]. Coded individual Fitbit user accounts were created for study purposes for each participant before the distribution of the devices. The accounts were linked to the iCardia platform to enable study investigators to remotely collect and monitor all Fitbit data from study participants in near real time. Participants received in-person training and printed instructions on how to perform the following tasks: (1) sync the activity tracker with the app, (2) view PA data on the app and tracker, and (3) charge the tracker at the initial group meeting. At the subsequent group meetings, this information was reviewed with participants upon request.

### Physical Activity Goal Setting

Participants received an initial step goal in the first week of the intervention based on their average daily steps recorded during baseline (week 0) with the ActiGraph accelerometer (ActiGraph GT3X+). At every group session, the step goals were updated based on participants’ average daily Fitbit steps in the period between each group session. The step goal was based on the mean weekly step count for the weeks before the study visit. We based our goal setting on the Women’s Lifestyle Physical Activity Program. The goal of that program was to increase each woman’s PA above baseline by at least 3000 steps per day, approximately 30 min of PA, over 24 weeks [19,34]. This is equivalent to 145 steps per day to achieve the program’s goal of 3000 above the baseline over 24 weeks. Therefore, the goal of this study was to increase, on average, 150 steps per day for each week between the study visits. For example, there were 3 weeks between group sessions 1 and 2, so the step goal the participant received in group session 2 was the mean of the 3 weeks plus 450 (150 steps × 3 weeks). The first step goal at visit 1 was set based on baseline step count captured by the ActiGraph, and the second and third step goals were set based on step count data captured by the Fitbit Charge HR. If the step goal was met, the participants were encouraged to increase their walking intensity. If not met, the step goal was the same as the previous group session.

### In-Person Group Sessions

The intervention comprised 3 in-person group sessions, each lasting 2 hours, held at a Chicago Park District location. At the first group session, participants were given back their Fitbit Charge HR and were trained on how to use the device and app. They also received an asthma education session and an introduction to the walking program. The other 2 in-person group meetings occurred approximately 3 weeks apart and included a short video on overcoming barriers to engaging in PA and meeting PA goals. The video was followed by a group discussion led by a nurse interventionist.

### Text Messages

Text messages (Multimedia Appendix 3) were developed using existing literature on text messaging to monitor asthma symptoms, and to promote asthma medication adherence and walking [25,35,36]. The sample text messages were reviewed by 10 African American female patients with asthma and by the nurse interventionist for the study, an African American woman with asthma, for further cultural modification before the start of the study. The text messages were at a fourth-grade reading level (measured by the Flesch-Kincaid Grade Level test) to account for differing education levels of women in the study.

Study participants received up to 5 text messages per week via the iCardia platform during the 7-week intervention. Messages containing personalized weekly step reports, educational content on asthma, and motivational/inspirational content to be physically active were sent to all participants 3 times a week (Monday, Wednesday, and Friday). An additional 1 to 2 messages per week were sent to remind participants of upcoming group sessions and to charge/sync the Fitbit device when applicable. The SMSs were reviewed by members of the research team each week and sent by the same research assistant throughout the entire study. The personalized weekly step reports were sent on Mondays and included a short report about their PA performance from the previous week praising the participants for meeting their step goal or encouraging them to achieve the step goal if not met. The nature of these text messages was adapted based on Fitbit data from the previous week and the progress of each participant. The educational text messages were sent on Wednesdays, were related to pollution and pollen warnings, and included tips on how to be more physically active and exercise, taking into consideration weather conditions and asthma. Participants also received 1 inspirational message each Friday. All participants received the same educational and inspirational messages each week.

### Feasibility and Outcome Measures

The feasibility of the intervention was assessed in the domains of recruitment capability, acceptability/suitability (adherence, retention, engagement, text messaging, acceptability, and complaints/concerns), and preliminary outcome effects on PA behavior (change in steps, duration, intensity, sedentary time, and adjusted sedentary time) and asthma measures (control and quality of life). These metrics, assessment strategy, and methods are summarized in Table 1. A Fitbit-based adjusted sedentary time measure was automatically calculated by iCardia to distinguish the minutes of true sedentary behavior from those minutes that users were simply not wearing their device. Fitbit classifies *sedentary* when MET values are ≤1, but this does not account for device wear time. Using wear time data as measured by heart rate, iCardia calculates an adjusted sedentary time by summing the number of Fitbit-based sedentary minutes when heart rate was nonmissing. Subsequently, we assessed differences between these 2 measures to determine the feasibility of measuring adjusted sedentary time, and also to determine the degree to which nonwear time impacts the actual Fitbit sedentary time measure under free-living conditions.

### Data Analysis

We collected accelerometer raw data at 30 Hz and uploaded using the ActiLife software (version 6.13.3, ActiGraph) at baseline. We converted raw files to counts per minute using the normal filter. A minimum of 10 hours of wear time, for at least 3 days, was retained for data analysis [27]. Accelerometer data were processed and analyzed after sleep time was excluded. The data were downloaded as step count and activity counts, which represents processed accelerations summed to a 60-second epoch length. PA intensity cut points were not applied to accelerometer data. Descriptive statistics are presented as means (M), standard deviation (SD), and relative frequency (%). Effect sizes (*d*) were calculated using the Cohen formula for paired samples. We performed visual inspection and the Shapiro-Wilk test of normality, which depicted normal distribution of the data [37]. Therefore, to compare sedentary versus adjusted sedentary time between week 1 to week 7, we conducted paired *t* tests at 5% significance level. We used Microsoft Excel and SPSS version 23 (IBM) for statistical procedures.

## Results

### Participant Demographics and Baseline Characteristics

Participants included 7 overweight or obese (BMI 34 kg/m^2^, SD 10 kg/m^2^), middle-aged (48 years old, SD 11 years) women. Overall, the participants’ asthma was not controlled (ACQ≥1=uncontrolled) and had evidence of moderate airflow obstruction (forced expiratory volume in 1 second % predicted <70%) [40]. The mini-AQLQ revealed some impairment in all domains (symptoms, activity limitation, emotional function, and environment stimuli). The greatest impairment was found in the environment stimuli domain, which included questions about feeling bothered by dust, cigarette smoke, and weather/air pollution [39]. At baseline, the mean ISWT was 400 m, which is similar to what has been found in a study of severe asthma patients [41]. At baseline (Table 2), the mean activity step count per day of the participants, as measured by ActiGraph, was over 10,000 (10,482 steps/day, SD 3170 steps/day).

**Table 2:** Baseline characteristics.

Variable		Value, mean (SD)
Age		48.29 (11.22)
BMI		34.12 (9.68)
FEV_1_^a^ % predicted		65.14 (14.11)
ACQ^b^ score		1.85 (1.54)
ISWT^c^ distance		400.00 (116.04)
Steps (ActiGraph accelerometer)		10,481.85 (3169.75)
**Mini-AQLQ^d^**		
	Symptoms	5.22 (1.79)
	Activity limitation	5.32 (1.62)
	Emotional function	5.76 (1.19)
	Environment stimuli	4.42 (2.21)
	Overall AQLQ score	5.18 (1.55)

^a^FEV_1_: forced expiratory volume in 1 second.

^b^ACQ: Asthma Control Questionnaire.

^c^ISWT: incremental shuttle walk test.

^d^AQLQ: Asthma Quality of Life Questionnaire.

### Process Feasibility

#### Recruitment

**Figure 2:**
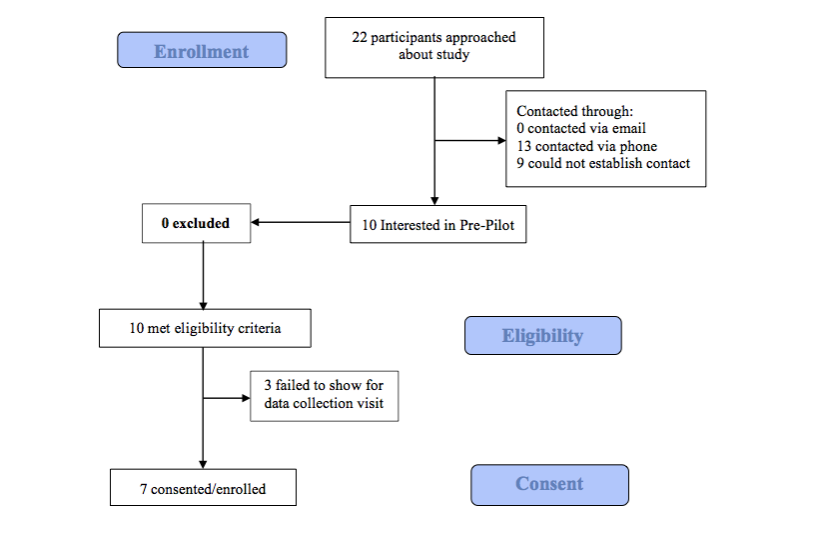
CONSORT flow diagram.

The Consolidated Standards of Reporting Trials (CONSORT) diagram in Figure 2 outlines the details of participant flow. A total of 22 asthma patients were contacted (in-person or by phone) to assess interest and eligibility in the study over 5 weeks. Of those, 10 eligible patients completed screening and offered enrollment; 3 participants did not attend the baseline visit, yielding a final sample of 7 asthma participants consented and enrolled in the study (Figure 2).

Barriers to recruitment included incorrect phone information from electronic medical record for potential participants, limited physician engagement in recommending the research program to their patients, recruitment only from the University of Illinois Health asthma specialty clinics, study staff availability to conduct study visits in the evenings, and space availability to accommodate for weekend appointments.

#### Feasibility: Acceptability and Suitability

#### Adherence to Group Sessions

Participant attendance rate for each of the 3 group sessions was 71% (5/7), 86% (6/7), and 86% (6/7), respectively. The overall mean participant adherence for all the group sessions was 81%. Reasons for not attending the group sessions included family obligations and lack of transportation.

#### Adherence to Wearing the Fitbit

**Figure 3:**
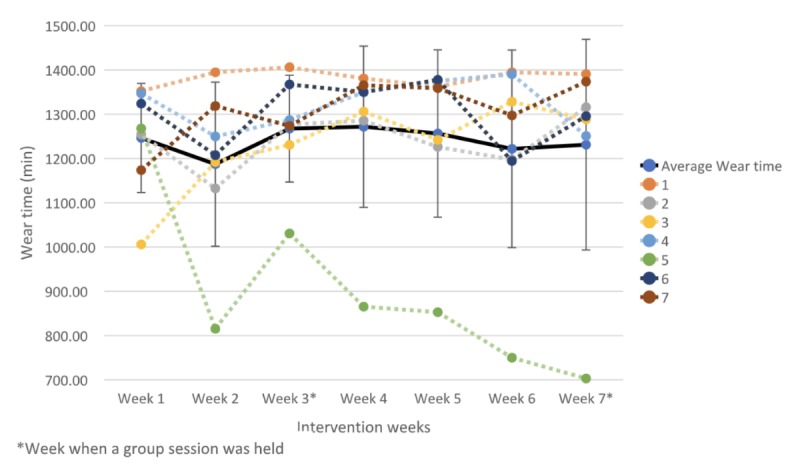
Mean, standard deviation, and individual Fitbit wear time over the 7-week intervention period.

During the 7-week intervention period, all participants adhered to wearing their Fitbit device for more than 600 min per day (valid day) most of the days, exceeding the minimum threshold for daily adherence. The overall mean fluctuated from 1247 (SD 123) min in the first week of the intervention, with a peak of 1272 (SD 183) min in week 4, and small decrease (1231 min, SD 238 min) in the last week of the intervention (Figure 3). Participants had on average 43.71 days of valid days (89.2%).

#### Fitbit Step Goal Adherence

**Figure 4:**
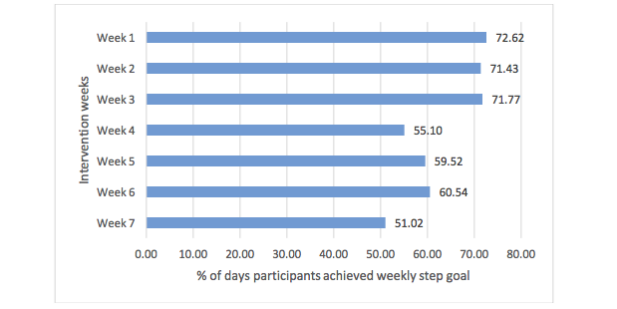
Mean weekly goal achievement over the 7-week intervention period.

The step goal was met on more than 70% of the days in the first 3 weeks for all participants. From week 4 to 7, the percentage of days the participants met their step goals decreased to just above 50% (Figure 4).

#### Retention

The retention rate was based on the number of participants who completed the postintervention evaluation subtracted from those who were enrolled in the intervention. Only 1 of the 7 women were unable to complete the postintervention evaluation in-person but completed the evaluation over the phone (retention rate=100%). This participant was categorized into the group who wore the Fitbit device during the intervention and received SMS but attended <2 group sessions. This participant only attended 1 group session and indicated that she was unable to attend the group sessions because of competing family commitments.

#### Engagement

The women that attended the group sessions were very engaged (1=not at all engaged to 7=very engaged) based on their engagement scores determined by the nurse interventionist. The mean engagement for each group session was 7 (SD 0), 6.8 (SD 0.4), and 6.8 (SD 0.4), respectively.

#### Text Messages Delivery and Content Appropriateness

A total of 217 text messages were successfully delivered to the participants over the 7 weeks. Each participant received an average of 31 (SD 1.3) messages over the intervention period, averaging 4.4 (SD 0.2) messages per week. The frequency of text messages was considered enough by 6 participants, although 1 participant mentioned she would have liked more, and indicated that 10 SMSs per week would be enough. All participants indicated the content of the messages was very appropriate or appropriate.

#### Acceptability

All 7 participants provided their input on acceptability of the intervention components (location, time, text messages, and Fitbit device and app) and the overall intervention at the completion of the last group session. All participants were very satisfied/satisfied with the text messages and with the Fitbit Charge HR. All participants felt very comfortable/comfortable charging and syncing the device with the app. All the participants were also comfortable using the Fitbit mobile app to view their data. None of the participants mentioned being unsatisfied with any of the aspects of the program. Most participants were satisfied/very satisfied with the location (6/7, 86%), time (6/7, 86%), and the intervention program as a whole (7/7, 100%).

#### Complaints and Concerns

Only 1 participant expressed that she expected more organized walking during the group sessions.

#### Scientific Outcomes: Safety, Compliance, and Treatment Effect

##### Safety

No serious adverse events were reported during the intervention, although 1 participant noticed “a little skin irritation” because of the Fitbit device use. She indicated that her wrist just needed some “air time,” which resolved the irritation.

#### Preliminary Outcomes Effects

### Fitbit Steps

**Figure 5:**
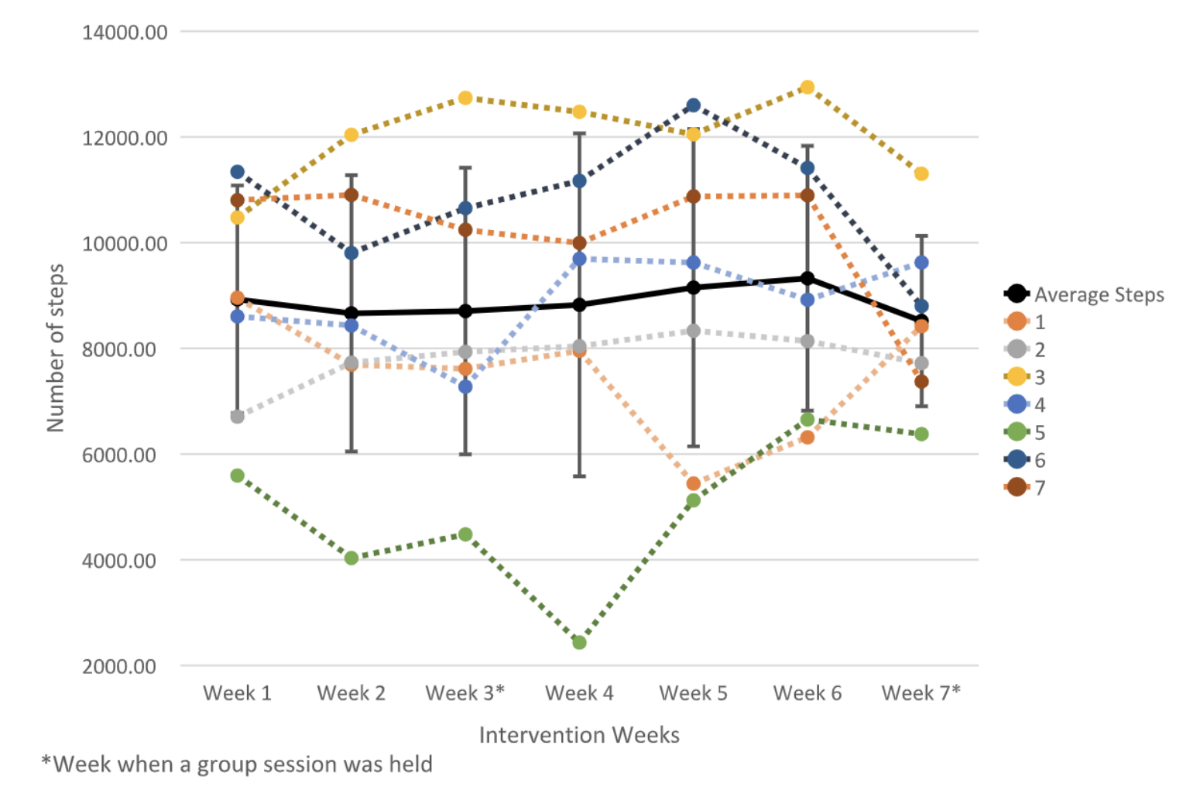
Mean weekly goal achievement over the 7-week intervention period.

Participants had a mean step count of 8926 (SD 2156) steps per day after the end of the first week of intervention. At the end of week 3, participants had maintained or increased their step goal. After the participants received their updated step goal, a slight increase in daily step count was observed from week 4 (8705, SD 2712) to week 6 (9325, SD 2505). In the last week of the intervention, only 2 of the 7 participants (#1 and 4) increased their daily step count. The other 5 participants (#2, 3, 5, 6, and 7) decreased their daily step count by 275 to 3522 steps per day (see Figure 5). An effect size calculation showed a small, not statistically significant, negative effect of the 7-week intervention on steps count (Cohen *d*=0.28, 95% CI −876.9 to 58.9).

### Fitbit Moderate-to-Vigorous Physical Activity

**Figure 6:**
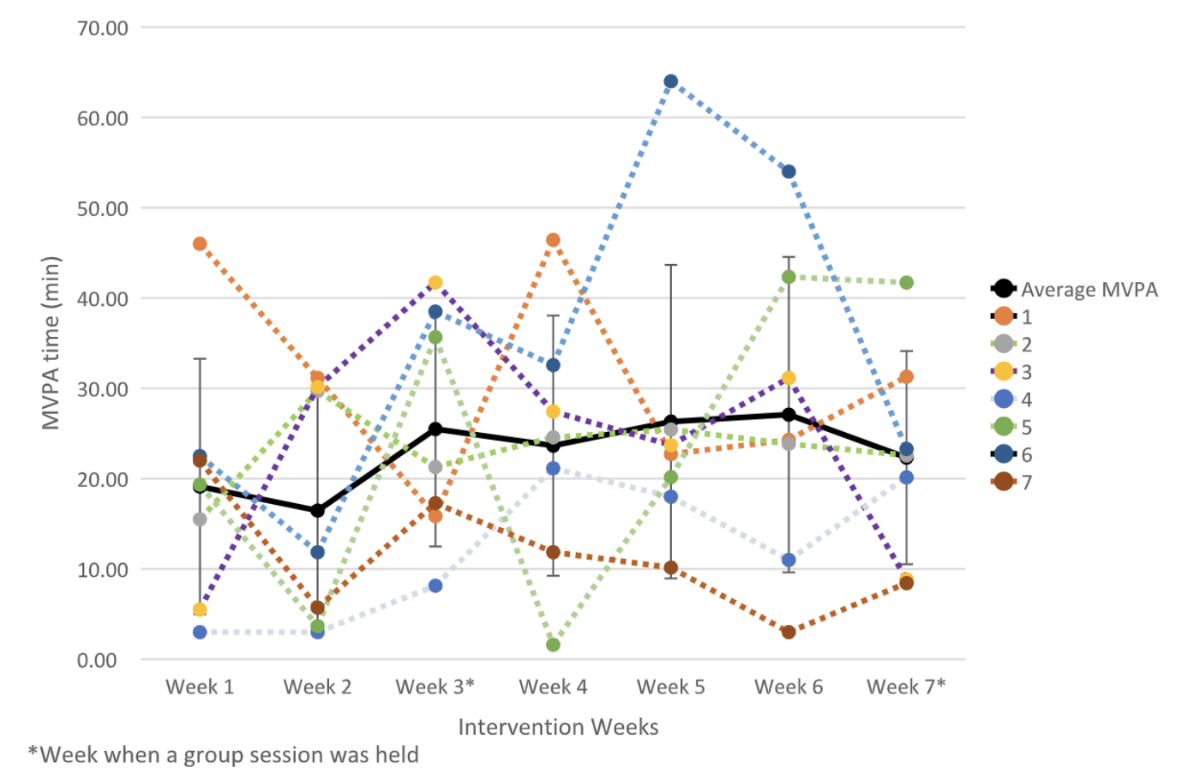
Weekly mean, standard deviation, and individual time spent in MVPA over the 7-week intervention period.

In general, we observed an increase in MVPA. After the first week of intervention, the participants averaged 19 (SD 14.15) min per day of MVPA. At the end of the last week, the time spent in MVPA averaged 22 (SD 12) min per day. We detected a small positive effect of the 7-week intervention on MVPA (Cohen *d*=0.24, 95% CI 0.1 to 6.4). All participants showed fluctuation in time spent on MVPA, with accentuated increases followed by steep decreases (Figure 6).

### Fitbit Sedentary Time

**Figure 7:**
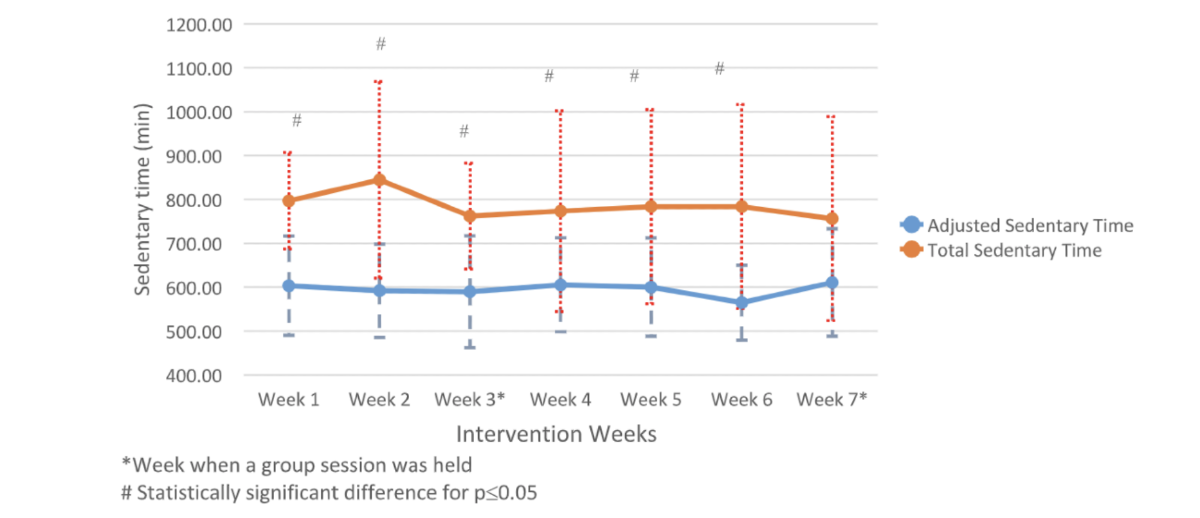
Comparison of the weekly mean time spent in total and adjusted sedentary behavior over the 7-week intervention period.

Participants showed a mean adjusted sedentary time of 595 (SD 110) min per week, with a peak of sedentary time during the last week of intervention (611 min/week, SD 122 min/week; Figure 7). We observed a statistically significant difference between the total and adjusted sedentary time in weeks 1, 2, 3, 4, 5, and 6 (week 1: Cohen *d*=−1.73, *P*=.006; week 2: Cohen *d*=−1.26, *P*=.01; week 3: Cohen *d*=−1.39, *P*=.009; week 4: Cohen *d*=−0.80, *P*=.049; week 5: Cohen *d*=−0.95, *P*=.04; week 6: Cohen *d*=−1.23, *P*=.04).

## Discussion

### Principal Findings

This study reports on the feasibility (process, acceptability, and scientific outcomes) of a walking intervention adapted for urban, low-active African American women with asthma. Our recruitment rate of 32% (7/22) is similar to what has been reported by another PA intervention in adults with asthma (32%) [13]. Although many of the recruitment barriers we encountered were similar to what has been reported in other clinical trials (incorrect patient contact information, limited physician engagement, and study staff and space availability to accommodate for evening and weekend visits), these issues are often augmented in low-income minority populations [42]. Frequent changes in phone numbers, an inability to take off from work, or childcare responsibilities may be contributing factors, and a multipronged approach is needed to overcome these recruitment barriers [43,44]. In future pilot testing, we will obtain multiple numbers of contact for potential participants, adjust study staff hours to accommodate for off-hour visits, and consider offering childcare during study visits. To address the physician engagement barrier, we plan to introduce our study at internal medicine and pulmonary faculty meetings and provide in-person reminders to physicians before seeing asthma patients in clinics.

In our outcomes of resource feasibility (acceptability and suitability), we had a high retention rate with no dropouts. In previous PA interventions in adults with asthma, 2 studies had dropout rates between 19% and 25%. Both studies had a similar intervention duration (8-weeks) to our study. One of them required attendance of 3 high-intensity interval exercise sessions per week in a hospital setting [45]; the other one included 6 group educational sessions, and a minimum of 20 home-based sessions of exercise training, during the 2-month study period [46]. Both studies found that younger employed adults were more likely to drop out. Our study included middle-aged adults, some of which were employed, and others were not. The less intensive group schedule that accounted for weekday workers and lifestyle-based PA program of our study may have contributed to our lack of dropouts, high participant engagement, and overall acceptability. Additionally, it was easier to coordinate and accommodate the schedules of a smaller group of women. The overall adherence to the intervention (group sessions and Fitbit device wear time) was high. The wear time was high likely because of our ability to remotely monitor participants’ adherence to wearing their Fitbit device regularly and address any adherence issues promptly using personalized text message reminders and behavior prompts. Most of the days throughout the intervention period were valid data, given the average daily wear time was well-over 600 min, which is often used in interventional studies as a cutoff point for determining a valid day of PA assessment [47]. Previous studies have shown self-reported wear time to decrease over time [48]. We saw high levels of adherence to the step goals during the first half of the study, which began to decline gradually in the second half of the intervention. These fluctuations in PA have been found in other PA interventions [48].

The scientific outcomes we assessed included the safety, compliance, and treatment effect of the intervention. Similar to other PA interventions in uncontrolled asthma patients, we did not have any adverse events, which is encouraging for future studies [45,49]. During baseline data collection using accelerometers, we found that the baseline step count in our study population of African American women with asthma was above 10,000 steps per day. This value was higher than expected on the basis of previous literature on urban African American women [19]. Despite the high step counts, participants were not engaging with the recommended amounts of MVPA weekly. Thus, our self-reported screening tool regarding MVPA levels was concordant with the objective measurements of MVPA. A seasonal effect may have occurred as the study was conducted in the summer months when more time is spent doing PA [50,51]. Many of the participants came from a convenience sample of the specialty asthma clinics, which may not represent the general asthma population. Although the accelerometer did not provide a screen for participants to see their step count, the participants may have been more aware of their PA since they knew it was being measured. Our small sample size did not allow for further analysis of other contributing factors to the high baseline step counts. During the intervention phase, the mean step counts captured by the Fitbit Charge HR in some participants exceeded 10,000; yet, much of their PA was of low intensity, not moderate-to-vigorous intensity, and thus met our eligibility criteria of <150 min of MVPA per week. We did see an increase in mean MVPA from just under 20 min per day at week 1 to 27 min per day at week 6, which declined to 22 min per day by week 7. Fluctuations in PA during PA interventions have been reported previously [52,53]. Sweet et al [52] found intrinsic motivation and identified regulation to be positively associated with PA. Other factors, such as stress and depression symptoms, may cause fluctuations in PA and were not assessed in this intervention [54,55]. Future studies should consider focusing on increasing the intensity of PA and better understand fluctuations in PA in this population.

One unique finding we found was in sedentary time measurements. An important issue with Fitbit devices and other wearable activity tracker devices that use proprietary algorithms to calculate activity measures is the misclassification of nonwear time minutes as sedentary time. Our study indicates that this can be a significant problem in research studies using Fitbit devices and may lead to spurious results. Over 7 weeks, there was an important and statistically significant difference between the number of sedentary minutes produced by Fitbit and the adjusted sedentary minutes calculated by iCardia taking into consideration participants’ nonwear time minutes. To the best of our knowledge, this is the first study that provides evidence on the feasibility of using real-time data from the Fitbit heart rate sensor to produce a wear time measure and subsequently adjust the number of Fitbit-based sedentary minutes. Future studies should further explore and validate this approach. Also, companies such as Fitbit, which produce consumer-based activity trackers, should leverage the ability of capacitive touch technologies to automatically and reliably detect when a wrist-worn device has been removed. This will facilitate compliance monitoring and improve accuracy of sedentary time measurement.

### Limitations

Limitations of this study include small sample size, short duration of the follow-up period, use of a general step goal for all participants, and lack of postintervention data collection. Additionally, our findings may not be generalizable to all low-active African American women as they did have a high baseline step count. However, this 7-week proof-of-concept study was focused on the feasibility of the modified PA intervention using mHealth tools to increase PA among African American women with asthma. We have identified several changes that need to be made in our recruitment and data collection methods and intervention delivery. The information gained from this study has helped us further refine the intervention to promote greater increases in PA and address study feasibility in a larger and longer pilot randomized controlled study, which is ongoing (ClinicalTrials.gov #NCT03265665).

### Conclusions

This study suggested that our modified PA intervention is safe and highly acceptable by African American women with uncontrolled asthma. These findings support the conduct of additional pilot testing to assess the feasibility of our modified intervention. We are conducting a 6-month randomized controlled PA intervention with 80 African American women with asthma, which includes the use of mHealth tools (Fitbit and iCardia for PA monitoring and text messages), step goals, group sessions that incorporate longer walking sessions, and asthma education (ClinicalTrials.gov # NCT03265665). Further work in this area will include evaluating the impact of motivational text messages on PA outcomes and assessing the efficacy and implementation potential of this walking program for African American women with asthma more broadly and in different settings.

## Appendix

Multimedia Appendix 1 ACTION (physicAl aCtiviTy In minOrity womeN with asthma) Pre-pilot Intervention Components.

Multimedia Appendix 2 Study Design for ACTION Pre-pilot.

Multimedia Appendix 3 Examples of text messages sent to participants.

## Abbreviations

ACQ: Asthma Control Questionnaire
API: application programming interface
AQLQ: Asthma Quality of Life Questionnaire
ISWT: incremental shuttle walk test
MET: metabolic equivalent
mHealth: mobile health
MVPA: moderate-to-vigorous physical activity
OR: odds ratio
PA: physical activity
