# Supplementary material for: A Walking Intervention Supplemented With Mobile Health Technology in Low-Active Urban African American Women With Asthma: Proof-of-Concept Study
Source: JMIR Form Res. 2020 Mar 11;4(3):e13900. doi: 10.2196/13900 (PMC7101169; doi:10.2196/13900)
Supplement: Multimedia Appendix 1 [file formative_v4i3e13900_app1.docx]

| **Supplemental Table 1. ACTION (physicAl aCtiviTy In minOrity womeN with asthma) Pre-pilot Intervention Components** |
| --- |
| - **One-time interactive group training/education session with asthma educator**    - Review benefits of physical activity in asthma   - Discuss strategies for preventing exercise-induced asthma |
| - **Fitbit Charge HR wearable monitor and training session**   - 15-minute training session on features of the device, device set-up, syncing and charging |
| - **Text messages (3-5 times/week)**   - Reminders on walking safety/asthma triggers (pollen/weather)   - Motivational and inspirational messages   - Personalized weekly step reports |
| - **Group meetings with a trained nurse interventionist**    - Receive individualized feedback on step goals   - Watch and discuss videos of African American women sharing experiences engaging in physical activity   - Engage in problem solving to overcome barriers   - Engage in walking/stretching   - Reinforce benefits of physical activity in asthma   - Peer interaction with other African American women with asthma |
| - **Intervention manual**   - Information on Fitbit Charge HR, how/where to walk and reinforces the benefits of physical activity in asthma. |
