# Supplementary figures and images for: A Walking Intervention Supplemented With Mobile Health Technology in Low-Active Urban African American Women With Asthma: Proof-of-Concept Study
Source: JMIR Form Res. 2020 Mar 11;4(3):e13900. doi: 10.2196/13900 (PMC7101169; doi:10.2196/13900)

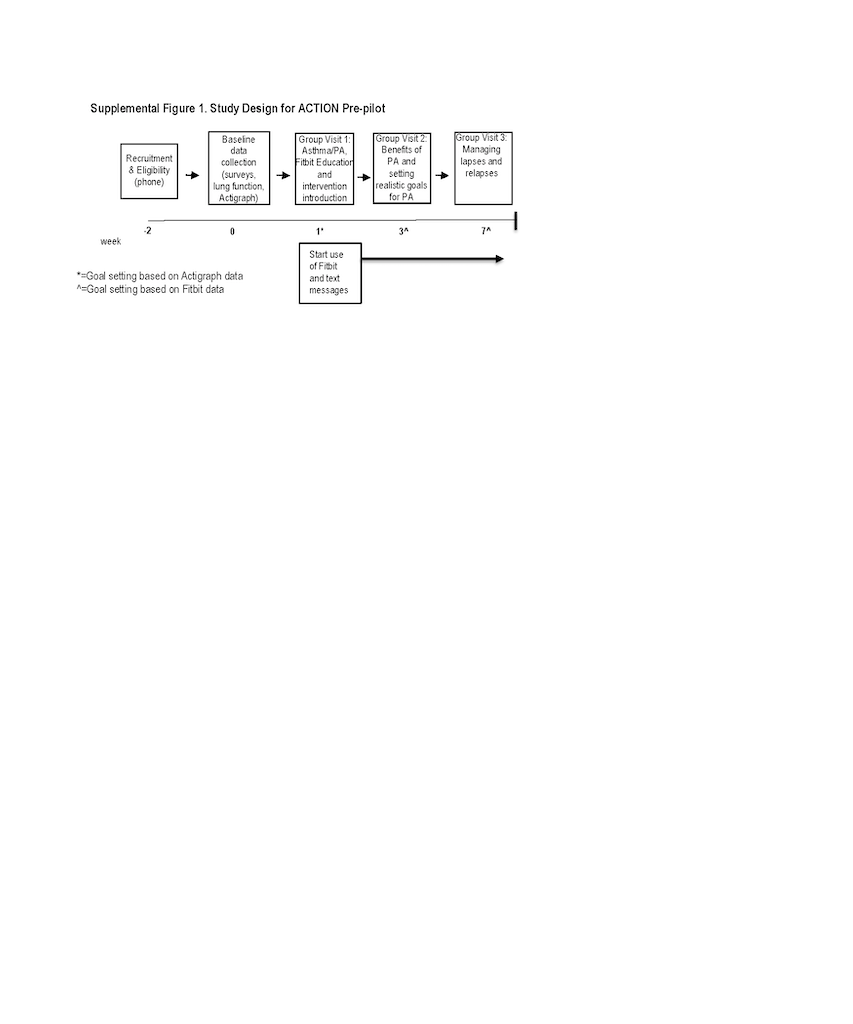

Supplement: Multimedia Appendix 2 [file formative_v4i3e13900_app2.png]
