# Supplementary material for: A Walking Intervention Supplemented With Mobile Health Technology in Low-Active Urban African American Women With Asthma: Proof-of-Concept Study
Source: JMIR Form Res. 2020 Mar 11;4(3):e13900. doi: 10.2196/13900 (PMC7101169; doi:10.2196/13900)
Supplement: Multimedia Appendix 3 [file formative_v4i3e13900_app3.docx]

Supplement Table 2. Examples of text-messages sent to participants.

| **Topic** | **Content** |
| --- | --- |
| Asthma | BE CAREFUL! The air quality today is unhealthy for your asthma. Reduce prolonged or heavy outdoor exertion. Take more breaks, do less intense activities. Watch for symptoms such as coughing or shortness of breath. Make sure you have your quick 911 relief inhaler with you if you walk outside. |
|  | Does asthma bother you when you go out for a walk? Try taking 1-2 puffs of your quick relief inhaler a few minutes before you go out. That should keep your breathing fine. |
|  | It is hard to remember – especially if you are feeling good, BUT it is a safe idea to have your rescue inhaler handy whenever you go walking outside of the house |
|  | BE CAREFUL –the pollution level is high today. It might trigger your asthma. Make sure you have your quick 911 relief inhaler with you if you walk outside. |
| Walking | Today is a great day for a walk! Have you tried walking with a friend or family member? |
|  | Today is a great day to walk! Don’t forget to stretch BEFORE and AFTER your walk and remember to keep yourself hydrated by drinking lots of water! |
|  | You are doing great! Keep on finding opportunities to walk more! Take breaks during work, park further away in parking lots! Go to the mall and walk! |
|  | You may not be able to walk 30 minutes a day every day but every little bit counts. Start small the minutes will add up!!. |
|  | Walking and talking can be a fun activity. Think of someone you know who might want to join you. |
|  | Today is a cold day. Try to walk inside or wear a scarf over your nose and mouth if you walk outside. |
| Asthma and Exercise | Worried about having an asthma attack when you walk? Try 1-2 puffs of your quick relief inhaler before you start. Do some stretches to get your body ready to go |
|  | Warm yourself up by stretching your arms and legs before you walk. This helps your lungs get ready to work and can decrease the chances of you having an asthma attack. |
|  | You may not believe it, but walking every day can actually improve your asthma. |
|  | Here is a little trick –if you breathe thru your nose when you are walking, it decreases the chance of triggering an asthma attack. Did you know that already? |
| Fitbit | We are missing you. It is been a while since the last time you synced your Fitbit. Please, remember to sync at every two days. |
|  | Your Fitbit battery level is low. Please, charge your Fitbit before your battery dies. Plan to charge it when you take it off before your shower or bath. |
|  | It seems that you gave a rest to your wrist yesterday. It’s ok doing it, but try to wear your Fitbit for at least 10h per day. |
| Step goal | You did a good job achieving your goal X days last week. What about trying to achieve your step goal more than X days this coming week? Keep your feet moving!!  Have a great week! |
|  | Oh no... You fell short yesterday on steps. Today is a new day. Try to make your step goal today! Keep on keeping on! |
|  | Hello! That’s the way to go! You did a good job achieving your steps goal over the weekend! Keep going! |
|  | How is your day going? You might want to walk more today. |
| Inspirational | I hope you are having a good day! Stop for a minute and think about something that makes you smile. |
|  | The secret to getting ahead is getting started. |
|  | It does not matter how slowly you go, as long as you do not stop. |
|  | Your desire to change must be greater than your desire to remain the same. |
|  | Always focus on how far you have come, rather than how far you have left to go. |
|  | When you feel like quitting, think about WHY you started! |
